# Deep learning facilitates multi-data type analysis and predictive biomarker discovery in cancer precision medicine

**DOI:** 10.1016/j.csbj.2023.01.043

**Published:** 2023-01-31

**Authors:** Vivek Bhakta Mathema, Partho Sen, Santosh Lamichhane, Matej Orešič, Sakda Khoomrung

**Affiliations:** a Metabolomics and Systems Biology, Department of Biochemistry, Faculty of Medicine Siriraj Hospital, Mahidol University, Bangkok 10700, Thailand; b Siriraj Metabolomics and Phenomics Center, Faculty of Medicine Siriraj Hospital, Mahidol University, Bangkok 10700, Thailand; c Turku Bioscience Centre, University of Turku and Åbo Akademi University, 20520 Turku, Finland; d School of Medical Sciences, Örebro University, 702 81 Örebro, Sweden; e Center of Excellence for Innovation in Chemistry (PERCH-CIC), Faculty of Science, Mahidol University, Bangkok, Thailand

**Keywords:** Cancer, Deep learning, Reinforcement learning, Precision medicine, Oncogene, Systems medicine

## Abstract

Cancer progression is linked to gene-environment interactions that alter cellular homeostasis. The use of biomarkers as early indicators of disease manifestation and progression can substantially improve diagnosis and treatment. Large omics datasets generated by high-throughput profiling technologies, such as microarrays, RNA sequencing, whole-genome shotgun sequencing, nuclear magnetic resonance, and mass spectrometry, have enabled data-driven biomarker discoveries. The identification of differentially expressed traits as molecular markers has traditionally relied on statistical techniques that are often limited to linear parametric modeling. The heterogeneity, epigenetic changes, and high degree of polymorphism observed in oncogenes demand biomarker-assisted personalized medication schemes. Deep learning (DL), a major subunit of machine learning (ML), has been increasingly utilized in recent years to investigate various diseases. The combination of ML/DL approaches for performance optimization across multi-omics datasets produces robust ensemble-learning prediction models, which are becoming useful in precision medicine. This review focuses on the recent development of ML/DL methods to provide integrative solutions in discovering cancer-related biomarkers, and their utilization in precision medicine.

## Graphical Abstract

**Figure:**
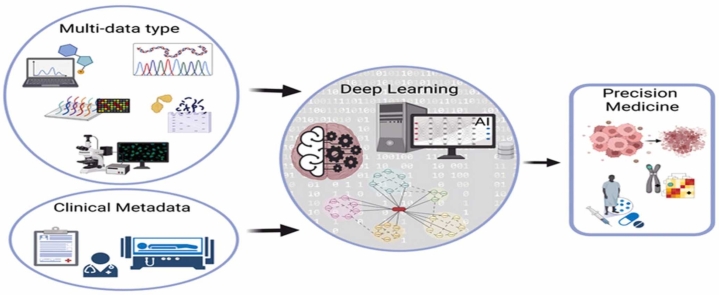
Deep learning facilitates the integration of various molecular data types and associated clinical metadata for biomarker discovery in cancer research.

## 1 Introduction

**Fig. 1:**
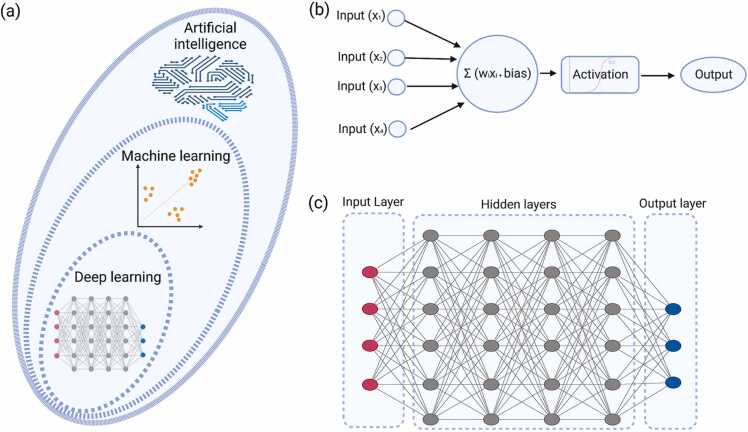
Basics of deep learning models. (a) Deep learning is a subset of the machine learning approach, which itself is a subdomain of artificial intelligence. The amount of dots in boundary line schematically represents amount of datasets available for model training (b) A basic unit of artificial neural network is a neuron, which can take input and undergo calculations involving weight and biases. The resulting values are subjected to an activation function to decide whether or not the neuron's input to the network is significant in the prediction process utilizing simpler mathematical procedures. (c) Deep learning models consist of multiple layers of such indivisual neurons with the ability to process high-dimension data. The dataset is fed into the network in the input layer, which is subsequently followed by multiple inner hidden layers, and final outcomes are computed on the outer layer with the utility of different activation functions.

Molecular biomarkers are physiological indicators that can indicate illness-associated alterations at the molecular level, assist in the prognosis of disease manifestation, and identify disease-related molecular targets [1], [2], [3]. In cancer pathology, the use of appropriate biomarkers for early diagnosis and prognosis is crucial to reducing mortality. Early diagnosis and prognosis of cancer are complicated by genetic polymorphisms, the presence of oncogenes, and epigenetic variables [4], [5]. Data integration strategies have aided patient clinical management in recent years by improving diagnostic accuracy and therapeutic efficacy [5]. In contrast to the intelligence of animals and people, artificial intelligence (AI) is the intelligence of machines that can perceive, synthesize, and infer knowledge. Machine learning (ML) is a kind of AI that can accurately predict outcomes based on training data without being specifically pre-configured to do so (Fig. 1a). The invention of the artificial neural network (ANN) allowed the modeling of complex non-linear systems by depicting the mechanisms of biological neurons. The ANN is made up of a network of connected units or nodes known as artificial neurons (Fig. 1b), which are modeled after neurons in the human brain. Each connection, similar to synapses in the human brain has the ability to transmit a signal to other nodes. An artificial neuron receives, analyzes, and communicates with other connected neurons. The output of each neuron is determined by some non-linear function of the sum of its inputs, and the "signal" is sent as a real number output. In particular, deep learning (DL) is a subset of ML approaches (Fig. 1a) that combine ANNs and representation learning. The learning can allow for highly dense and fully connected multi-layered networks that can be trained in supervised, semi-supervised, or unsupervised settings (Fig. 1c). These ANNs can also be used to construct autoencoders that use unsupervised learning techniques for data encoding.

Conventional polygenic risk scoring through the utilization of AI and ML is also becoming a promising tool for the early detection and prognosis of cancer [6], [7]. In this context, deep learning may provide a suitable alternative for modeling complex traits and incorporating previously restricted multidimensional medical imaging datasets [8], [9], [10], [11]. Although the conventional ML approach is the most efficient analysis technique in multiple medical discovery and clinical decision support systems (CDSS), the utility of DL is gaining attention in multi-data-type analysis involving ensemble-based disease study models [5], [12], [13]. Furthermore, DL-assisted identification of susceptible genes and associated proteomics and metabolomics profiles could be a useful strategy to detect cancers at early stages [5], [14], [15], [16], [17], [18]. Such an integrative strategy may also provide targets for precision medicine, which may help to enhance the prognosis for total recovery. Recent studies suggest that a DL-based approach that integrates a broad range of datasets, including histology, magnetic resonance imaging (MRI), X-rays, and chromatograms, along with multi-omics data can significantly improve the accuracy of diagnostic models for cancer [8], [19], [20]. Precision oncology requires the ability to predict outcomes for specific cancer patients, such as survival or metastasis [21]. DL approaches, such as graph neural networks (GNN), applied to gene regulation and metabolic pathways, are emerging as useful tools in the study of tumor metastasis [21], [22], [23]. GNN is a form of neural network that acts directly on the graph structure consisting of multiple nodes, each representing an entity. Node categorization is a common utility of GNN. Moreover, the development of generative DL models is currently being utilized for drug target discovery and *de novo* synthesis of investigational new drugs to facilitate cancer research [24], [25]. This review briefly explores the current trends in DL, which facilitate multi-data type analyses, biomarker discoveries, and precision medicine.

## 2 Deep learning-assisted medical imaging and multi-omics data analysis in cancer research for early cancer diagnosis

**Fig. 2:**
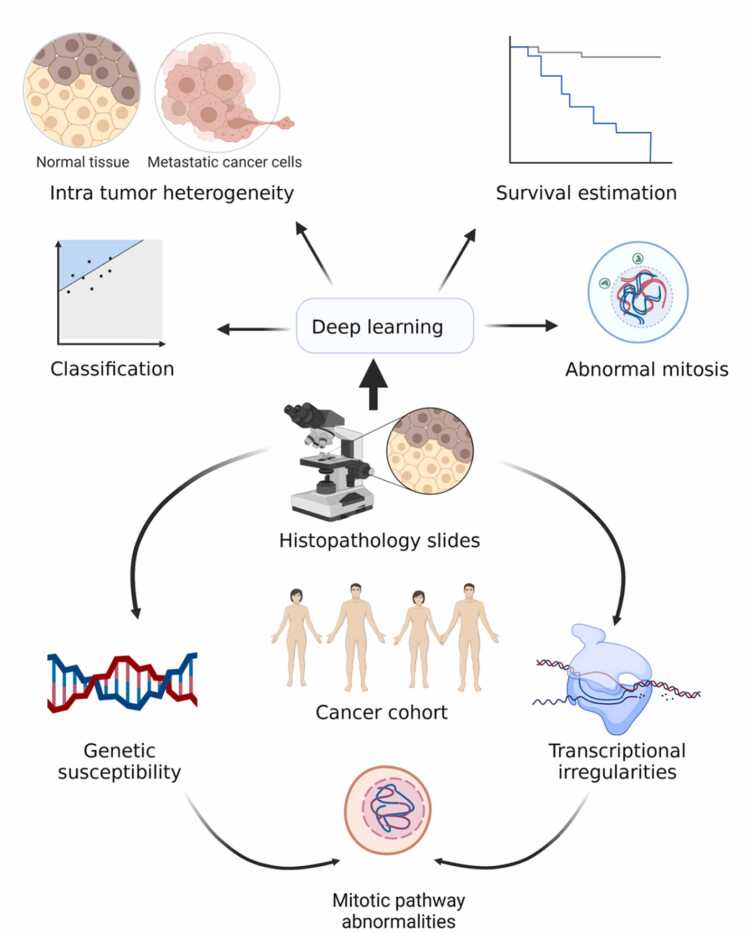
Integration of histological slides and genetic susceptibility data in deep learning techniques for malignancy prediction. Histological slides provide strong evidence related to clinical manifestations of cancer such as neoplasms, malignant tumors, and metastasis. Deep learning techniques can combine these traditional image-based datasets with well-known genomic tests to make a strong model for early cancer diagnosis that is much more accurate than individual tests.

Medical image categorization is essential in clinical diagnosis, prognosis, and educational activities [26], [27]. Imaging biomarkers are based on the anatomical, histological, or radiographic characteristics of samples. Histology slides are a valuable source in clinical practice for screening cancer biomarkers such as angiogenesis, tumor growth, and metastasis [28], [29]. Manual evaluation of histological slides (Fig. 2), X-ray, computed tomography (CT), and MRI are laborious and often subject to human error, leading to misdiagnosis. In recent years, DL has demonstrated exceptional accuracy in processing medical imaging data for disease diagnosis, including chest radiography, breast cancer screenings (or mammograms), and CT scans [8], [18], [30], [31]. The success of DL in medical imaging is driven mainly by the development of convolutional neural networks (CNNs), a class of deep neural networks (DNNs) that perform excellent visual imagery feature detection with high sensitivity and specificity [32]. 3D versions of CNN also assist in solving problems related to 3D image segmentation, which is necessary for categorizing brain tissues, heart structures, and abdominal organs [8], [33]. The transfer learning (TL) is a DL-based strategy that focuses on retaining information learned while addressing one issue and transferring it to resolve a similar but distinct problem. Well-established CNNs such as VGG16 [34] which have been trained to recognize a variety of objects, can be leveraged through TL and applied to relatively small datasets to yield highly accurate diagnosis models [35], [36]. Similarly, one-shot and few-shot learning models are also being used for semantic feature learning tasks to help diagnose lung cancer recurrences [37] and lymph node metastasis [38], [39]. However, the datasets used for training these models have a significant impact on their accuracy. Therefore, the quality of training datasets can highly influence the performance of models [40]. CNN can also detect features in non-image data such as gene expression *via* transforming non-image datasets into feature-rich image vectors. This can be subjected to a trained CNN classifier for cancer-type prediction based on gene expression data [41], [42]. Recent developments in DL include the swarm learning (SL) technique, which involves parties working together to build AI models, while avoiding data transfer and monopolistic data governance for large multi-centric datasets. Swarm learning was implemented on multi-centric datasets of histopathological images from over 5000 cases. It assisted in the prediction of BRAF protein mutation status and microsatellite instability directly from colorectal cancer histological slides [43]. Together, these studies suggest that DL is paving the way to improve early cancer diagnosis using medical images. Independent dataset validation is a commonly used technique in AL and DL for evaluating the performance of a trained model. This technique involves using a separate dataset for evaluation, which is isolated from the training and testing dataset, to ensure the generalization of model to unseen data. Independent dataset validation have been utilized to evaluate the performance of several DL models ranging from imaging biomarkers in neurodegenerative such as Alzheimer [44], [45], breast cancer [18], histopathological analysis for malaria [46] to time series-labelled tumor markers [47]. DL is an emerging filed, thus, it is important to note that due to the lack of standardized protocols for data collection and the limitations in technical capabilities, not all models or studies have the ability to utilize independent dataset validation. This can pose a challenge for ensuring the generalizability of model performance and should be taken into consideration when designing and evaluating deep learning models.

**Fig. 3:**
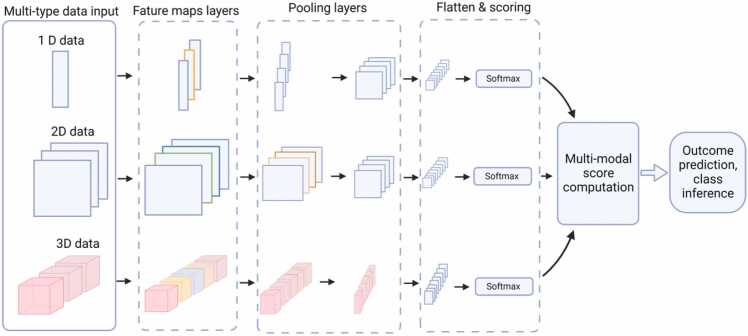
Schematics illustrating utility of simple multi-modal deep learning approach in cancer research. The multi-type data input can include blood metabolite concentrations (1D data), histological or X-ray images (2D data), and MRI or CT scan images (3D data). The data undergo feature mapping followed by multiple pooling operations within DNN. The layers are subsequently flattened, and a score is computed using a scoring function (e.g. softmax) for each model. These scores from multiple models are further processed to compute the final outcome as disease class prediction or inference.

DL may assist in the in-depth study of cancer by enabling the integrative analysis of multi-omics datasets [48], [49], [50], [51]. A generalized schema (Fig. 3) involving multi-type data input illustrates the utility of multi-model DL approach for data classification or inference. The schematics summarizes intake of hypothetical multi-dimension data such as: blood metabolite concentrations, histological images of biopsy, and CT scans to classify or inference possible outcomes of a cancer-related diagnosis. These input datasets can be of any type, such as X-ray images, blood metabolite concentrations, microarrays, and genomics data. Lee et al. integrated a DL-based autoencoding method with multi-omics datasets, including genomic, transcriptomic, and epigenomic (mRNA, miRNA, DNA methylation, and copy number variations) datasets obtained from patients with lung adenocarcinoma (LUAD). The model was able to learn representative features of the disease to differentiate the patient subgroups, showing the potential of DL in LUAD prognosis [48]. Multi-omics data associated with breast cancer have frequently been used in DL studies [18]. In a separate study, Zhang et al. applied DL methods by integrating gene expression, gene copy number variations, miRNA expression, and DNA methylation data to predict muscle-invasive bladder cancer (MIBC) [49]. This study identified the genomic and immunological differences between high- and low-risk patients. It also recognizes the role of Keratin 7 (*KRT7)* as a biomarker of MIBC. Recently, Hira et al. showed that DL-based variational autoencoders could be utilized for the classification of ovarian cancer transcriptional subtypes using multi-omics data from the cancer genome atlas (TCGA) datasets with model accuracies ranging from 87% to 93% [52]. A DL approach was also used in the identification of features linked to differential survival of patients with hepatocellular carcinoma (HCC) [53]. A DL-based survival-sensitive model was developed using RNA/miRNA sequencing and DNA methylation data obtained from patients with HCC in the TCGA database. The model stratified two optimal subgroups of patients with significant survival differences and was validated using five external datasets from various omics types. This study suggests that DL can be used for the prognosis of HCC [53]. Also, Poirion *et. al.* suggested a hybrid approach using a novel DL and ML framework called “DeepProg” which utilized multi-omics data to build a highly predictive model for patient survival risks among various cancer subtypes [5].

The promising outcomes of DL-based studies have accelerated broader interest in combining metabolomics and other omics data for similar applications, such as potential biomarker discovery [14], [53], [54], chemical structure identification [55], [56], and disease comorbidity analysis [57]. Although implementing a DL approach with genomic, DNA methylation, and transcriptomic data for biomarker prediction and cancer treatment selection is relatively common [54], [58], [59], the use of metabolomics-based biomarkers is gaining attention because of their direct relation to the cancer phenotype [15], [60], [61]. Nonetheless, one of the potential liabilities of using big multi-omics dataset is that it could diminish the representation of sparsely occurring diseases as the large percentage of frequently observed features in training database may create biasness in classifier models.

Furthermore, the benefits of such findings show that including multi-omics data in a DL model improves its accuracy when compared to using only a single omic dataset [48], [62]. Given the growing trend of applying DL in cancer research, modeling patient outcomes and biomarker discovery may facilitate timely diagnosis and improve the clinical management of patients with cancer.

## 3 *De novo* synthesis and prediction of compound properties and activities using deep learning approach

**Fig. 4:**
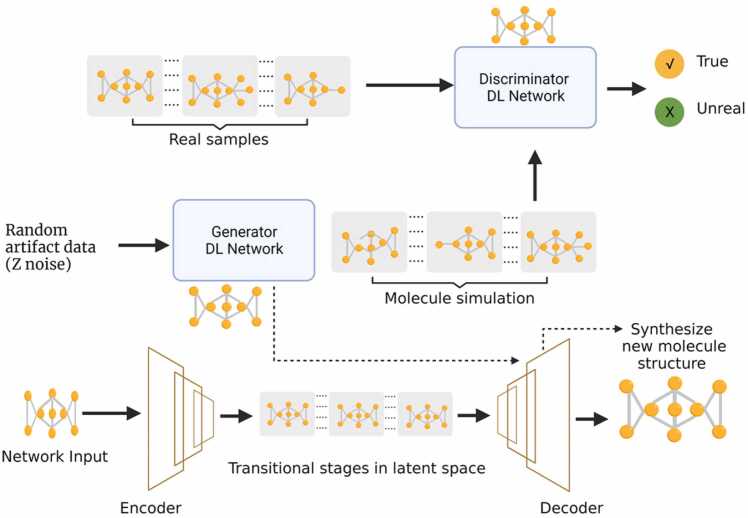
Schematics of *de novo* simulation of novel compound structure based on generative deep learning models. Unlike the use of SMILES to discover potential drug structures, the deep learning-based generative models can learn molecular features of drugs in a training dataset and simulate entirely new compounds within a similar distribution. This is achieved by combining GAN with a suitable autoencoder to provide optimum results. The autoencoder is first trained using a standard molecular database as network input. The trained encoder produces latent vectors that are used as the training input for GAN using feedback obtained from the discriminator network. The new compounds are simulated by the trained generator network of GAN using random Z-noise and then utilizing the decoder to transform the sampled latent vector into a chemical structure. The solid arrow represents the flow of training data in the network. The dotted arrow represents the flow of data in the optimized GAN and autoencoder network. GAN, generative adversarial network. SMILES is a simplified molecular-input line-entry system.

Drugs designed using synthesis-based predictions have a long history, dating back to the early 1960 s. It is exceedingly difficult to design novel compounds using conventional tools [25]. ML/DL approaches have shown significant potential in implementing the simplified molecular-input line-entry system (SMILES) as the basis for describing molecular structures [63], [64]. AI and generative models have recently been used in *de novo* drug design (DNDD) and compound optimization [25], [56]. The DNDD approach is a computational growth-based method that generates new chemical structures without prior knowledge of their structural and chemical properties. The synthesis is based on the creation of new chemical entities that meet a set of requirements by employing computational growth methods. Traditional methods require ligand- or structure-based designs that are based on the active site and binder of the target molecule [65]. Other molecular generation or *de novo* design techniques include inverse QSAR10 [25], [66], particle swarm optimization, and genetic algorithms [25], [67]. These methods generate new molecules with specific chemical properties based on the properties of training datasets. In recent years, generative models have been increasingly used in molecular *de novo* design owing to their ability to learn the characteristics of real-world training examples and to create entirely new synthetic entities with similar characteristics [68], [69]. A recent study utilized a generative adversarial network (GAN) model that integrated information from systems biology, molecular design, and transcriptome data to train a DL generative model that could autonomously design compounds with a high likelihood of imparting the desired transcriptome profile [70]. Fig. 4 shows a schematic of the recent developments in *de novo* compound design using generative models and autoencoders. Despite the lack of theoretical evidence from other ML approaches, the results suggest that a DL approach may hold potential comparable to well-established conventional ML and statistical methods in cancer studies [71], [72]. Recently, promising results were reported for a *de novo* molecular generation method using a latent-vector-based GAN that did not require SMILES for compound synthesis [24]. Similarly, DL approaches have been explored to assess the feasibility of drug repurposing in oncological studies encompassing genetic profiles and drug-phenotype associations [73], [74], [75]. In association with the conventional ML approach, a range of artificial networks such as GANs, recurrent neural networks (RNNs), and autoencoders have been effectively used to generate innovative *de novo* drug creation methodologies [56], [69]. Thus, DL-based generative models have the advantage of utilizing multi-type biological training dataset and are not limited to simple molecular descriptors like SMILES. These developments highlight the potential of DL for *de novo* synthesis and compound characteristics prediction for the development of cancer drugs.

## 4 Potentials of deep learning in tumor-associated metabolic pathways prediction and graph-based convolution neural networks in cancer studies

A recent study by *Petrovsky* et al. used 1D and 3D CNNs to differentiate patients with kidney, ovarian, and prostate cancers from healthy controls by analyzing metabolomics datasets [51]. The well-trained DL model was able to recognize several oncopathologies with an area under the receiver operating characteristic curve (AUROC) ≥ 0.95. Recently, Inglese et al. applied a DL approach to mass spectrometry (MS)-based 3D desorption electrospray ionization (DESI) imaging data to predict the heterogeneity of the metabolic phenotypes of cancer. This allowed unsupervised clustering of tumor tissue to identify subregions distinguished by the abundance of certain metabolites, revealing biological heterogeneity within the tumor [9]. In another study, DL was applied to metabolomics data to identify the metabolic features of complex traits, which accurately predicted the status of an estrogen receptor in breast cancer samples [15]. This study compared a feed-forward DL network with six ML techniques, namely random forest, support vector machine, recursive partitioning and regression trees (RPART), linear discriminant analysis, and generalized boosted models, in terms of their ability to classify negative and positive estrogen receptor breast cancer tissue samples. The DL approach achieved the highest accuracy (AUC = 0.93), outperforming well-established ML methods. Furthermore, the DL model uniquely identified eight novel metabolic pathways associated with breast cancer, paving the way for future investigation [15]. Recently, Kopylov et al. utilized a 1D CNN to recognize metabolic signatures that could distinguish between different cancer phenotypes and schizophrenia at the comorbidity level with a model accuracy of ≥ 90% [57]. They deployed a neural network to analyze complete mass spectra and were able to efficiently distinguish unrelated pathologies and a control (healthy donors) group with a model accuracy of 90%, and different subtypes of oncophenotypes with an accuracy of 94% [57]. Zhou et al. used an autoencoder-based DL to learn the latent representative features of MS data and thereby detected paragangliomas and ovarian cancer with accuracies> 95% [76]. Shaffie et al. implemented a DL autoencoder classifier framework using CT scan images and breath-based volatile metabolic marker data as inputs to achieve over 97.8% accuracy in lung cancer diagnosis [77]. Furthermore, DL models can facilitate network inference, including metabolite–metabolite interactions within a biochemical pathway. Fang et al. developed a DL algorithm *Lilikoi v2.0* [78] (an upgrade of *Lilikoi v1.0* [79]), which uses a multi-layer neural network trained with a stochastic gradient descent search. The *Lilikoi* algorithm can transform and incorporate metabolomics data from samples to generate personalized pathway profiles, which can aid downstream classification or development of a prognosis model based on the metabolites linked with the corresponding pathway [78]. The tool demonstrated marked alterations in the metabolic pathways associated with breast cancer in estrogen receptor-positive and -negative samples [15], [78].

Indeed, the application of DL in metabolomics and lipidomics has been relatively slow, mainly because of the scarcity of reference standards, accurate analytical methods, computational resources, expertise, and interpretability. Because DL models require large datasets for training, low sample sizes are likely to decrease the sensitivity and accuracy of DL models to predict metabolic phenotypes [14], [55], [80]. The limited number of labeled patient samples is a common issue for training any type of ML or DL model [81]. This, together with the significantly greater number of features describing each sample, negatively impacts unsupervised learning. An improved performance in the ML models while predicting individual patient responses to therapeutic drugs has been achieved by iteratively combining supervised models and feature selection [82], [83]. In the context of DL models applied to image datasets, few-shot classification models are gaining attention owing to their architectural characteristics to learn from smaller dataset sizes [33], [38]. Nonetheless, rapid advancements in high-throughput metabolomics, together with an efficient DL approach, might ameliorate these shortcomings.

A graph convolutional neural network (GCNN) is a type of deep neural network that works directly with a graph structure. A GCNN can be a DNN with fully connected convolutional layers. Node categorization is commonly used in the GCNN [23]. In essence, each node in the network has a label, and the task is to predict the labels of unknown nodes based on other node information. The GCNN model was developed as a more flexible way of propagating information through many tiers of neighboring nodes in a graph network. Traditional CNNs are inefficient in capturing complex neighborhood information because they assess small regions based on a fixed convolutional kernel, resulting in limited performance and interpretability of functional and structural feature analyses [22], [23]. ML models that employ a graph network have an advantage in that they can accurately describe complex physical entities and processes, as well as irregular interactions. A GCNN was used to conduct pan-cancer analysis to detect the likelihood of tumor metastasis. Gene regulatory networks and gene expression features were extracted using a GCNN. The high-dimensional features of each sample were represented as image pixels and utilized to predict the likelihood of tumor metastasis in each patient [84].

**Fig. 5:**
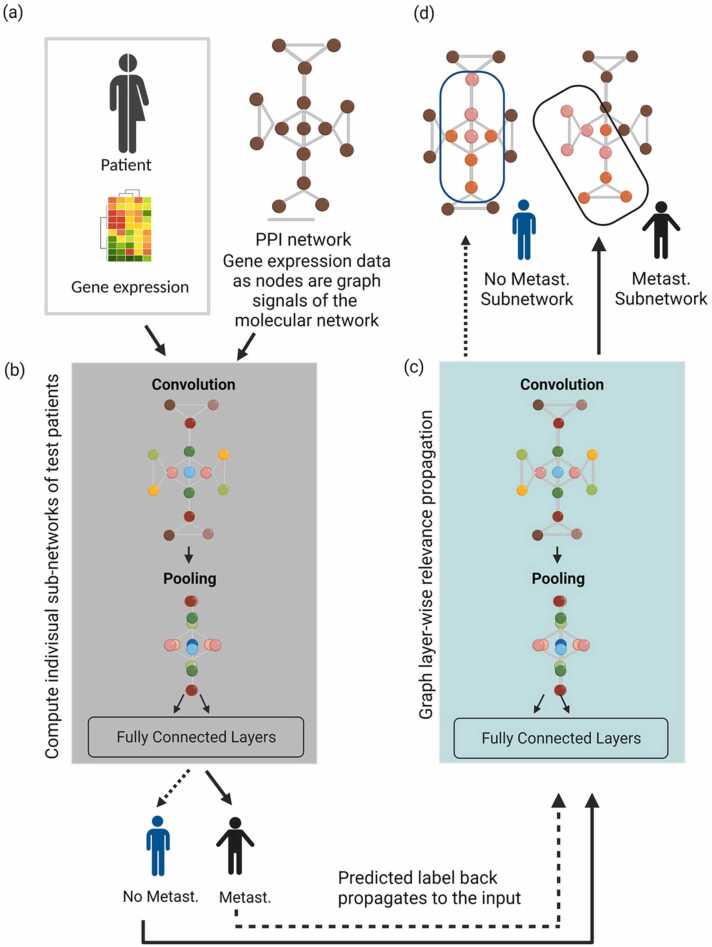
Schematics of a graph convolution neural network (GCNN) for cancer diagnosis. (a) The clinical data and gene expression or proteomics PPI datasets of cancer and control groups can be parsed to (b) a specialized GCNN deep learning model, which combines this information to create a graph network-based prediction model. The GCNN uses information from training sets to customize individual patient medication regimens and (c) generate patient-specific subnetworks to (d) assist in precision medicine therapy. The blue and black oval-cornered boxes in ‘d’ represent metastasis and non-metastasis gene-subnetwork identified by GCNN, respectively. Each circle in ‘a’ represents gene expression prior to normalization or differential analysis. Multi-colored circles ‘b-d’ schematically represent differentially expressed genes as nodes with varying intensities. Solid and dotted arrows represent information flow in GCNN for metastatic and non-metastatic groups, respectively. GCNN, Graph convolution neural network; Metast., metastasis; PPI, protein-protein interaction network.

Metastasis is the most common cause of cancer-related death. Monitoring variations in cancer gene expression is crucial for understanding metastatic pathways for tumors and cellular activities. A GCNN was recently used to combine the image features of metastasis to local lymph nodes for cancer trajectory prediction and clinical decision-making [21]. A GCNN can utilize the gene expression data of a metastatic and non-metastatic cohort as a graph signal molecular network and compute an individual subnetwork for a test patient cohort (Fig. 5). In summary, clinical metadata from the diseased and control groups (Fig. 5a) along with corresponding gene expression and/or other multi-omics data (Fig. 5b) as nodes, could be utilized to train a customized GCNN prediction model. This enabled the identification of patient-specific genes or feature subnetworks to deploy precision medicine (Fig. 5c-d). The differential expression of genes between training cohorts using cross-validation can optimize a GCNN model and identify the labels of network nodes, which correspond to potent biomarkers for metastasis (Fig. 5). Graph-CNN-based modeling presents an effective DL-based approach for selecting features, which take advantage of past information and generate patient-specific subnetworks, thereby influencing the outcome of individual categorization. For instance, a GCNN was employed to integrate patients’ clinical-genomic data for pan-cancer analysis and investigate the stratification of lung tumors for immunotherapy [85]. The GCNN has the advantage of flexibility in clinical metadata integration, which may include clinical subtypes as well as patient-specific genes that might be associated with malignant or benign phenotypes. A variation of GCNN was utilized to predict the effect of pharmacotherapy on *in vitro* cancer cell growth [22]. Correspondingly, by using a GCNN, researchers have been able to combine different features and extract cancer-related traits from gene expression data with significantly improved prediction accuracy [84]. Together, these results raise the possibility of using the GCNN as an effective DL method to examine the clinical effectiveness of cancer diagnostics and treatments.

## 5 Deep learning-based ensemble modeling for precision cancer medicine

**Fig. 6:**
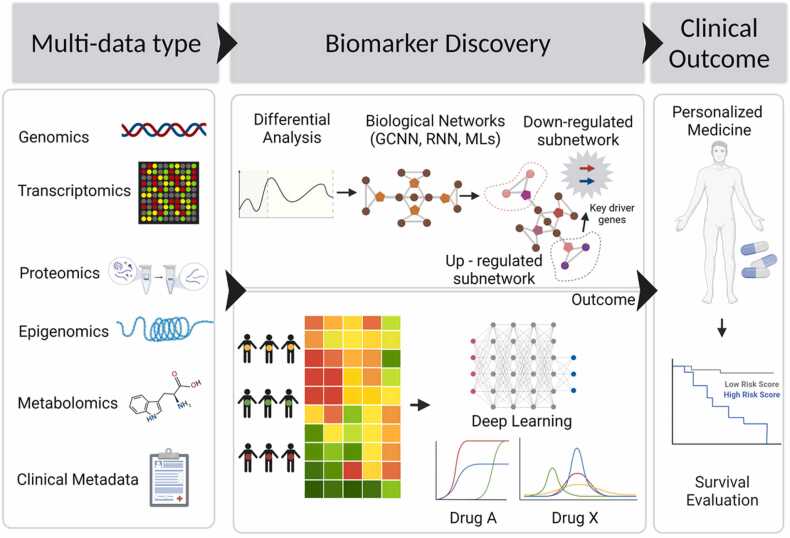
Deep learning ensemble modeling for precision medication. Ensemble modeling can combine multiple data types along with clinical metadata with a variety of conventional ML and DL models. It predicts the final scores based on the ‘voting’ of multiple models, which makes the results resilient to single-point model failure. Patients’ survival outcomes and treatment efficacy output for an investigational medication can be fed back to the ensemble model for optimization of precision medicine. GCNN, graph convolution neural network; RNN, recurrent neural network; ML, machine learning.

One of the major aims of cancer precision medicine is to identify the molecular traits responsible for certain clinical outcomes. At present, a strategy involving molecular traits through drug-specific informative genes remains a popular means to investigate sensitivity or resistance to cancer drugs [81]. DL approaches have a myriad of modeling architectures, making it difficult to determine the best approach. Occasionally, it might be feasible to undertake a systematic study of all possible gene candidates using microarray gene expression data to identify new potential biomarkers. However, feature selection based only on statistical processing and single data types often does not account for personalized molecular information and clinical metadata, resulting in the selection of genes or traits with limited biological relevance [86]. Recurrent neural network and long-short-term memory (LTMS) models are good at analyzing repetitive patterns in sequences and time-series forecasting, whereas CNNs are better at detecting image features [87], [88], [89]. When omics data must be computed together with histological data and other clinical metadata, it is reasonable to adopt a DL-based multi-model ensemble network for biomarker discovery. A previous study suggested that ensemble-based modeling performs well in breast cancer diagnosis, in terms of both stability and prediction accuracy because of the collective representation of multi-model prediction schemes [4]. A recent study in breast cancer patients implementing bimodal DNN with heterogeneous gene expression data and clinical information outperformed all prognosis prediction models, as indicated by survival analysis and model performance, with the potential for the development of precision medicine [86]. Rather than depending on single-model feature prediction, an unsupervised network-based selector combined with DL models may enhance robustness. This is especially important in precision medicine, where a patient’s clinical information might have a significant impact on one model, while having little or no effect on others. Fig. 6 summarizes how multi-omics data, along with clinical metadata, can be integrated into an ensemble-based multimodal network to identify potential biomarkers and facilitate precision medicine. Biomarker discovery involves an ensemble-based multi-omics feature selection strategy, and the efficacy of the predictor is evaluated on the basis of clinical survival analysis. This strategy is highly beneficial for precision medicine, as patients’ clinical metadata can be directly correlated to the outcome of survival analysis; thus, the best performing models within the ensemble will have more influence on biomarker discovery and disease prediction. Taken together, these studies suggest that DL-based ensemble modeling can be advantageous when single data-type modeling is unable to achieve high performance. A limitation of multi-omics ensemble-based models is that they are computationally intensive and, therefore, difficult to deploy in clinics. Additionally, the generation of multi-omics data requires sophisticated instruments, and the acquisition of erroneous data may occur owing to a lack of expertise, ultimately reducing the performance of ensemble models.

## 6 Combination of deep learning and deep reinforcement learning

DL models learn from a training dataset and then apply that knowledge to infer classes in an entirely new dataset, whereas reinforcement learning (RL) models learn dynamically using continuous feedback to optimize a predesignated reward [90], [91], [92]. Deep reinforcement learning (DRL) combines the features of both RL and DL models to achieve optimum results in solving computationally intensive tasks, such as real-world object recognition, molecular structure simulation, and gaming. DRL has been used for applications ranging from computer gaming to medical technologies by incorporating DL models into reinforcement learning algorithms [93], [94], [95]. NVIDIA, a well-known gaming technology company, is actively involved in the development of AI systems for medical research, which illustrates the growing interest in DL and DRL in this field [96], [97], [98]. In terms of innate immunity, Petersen et al. used DRL to develop an adaptive precision medicine policy that defines effective multi-cytokine therapy for sepsis patients [99]. Cancer-related omics datasets using DRL have been able to address a wide range of sequential decision-making problems, suggesting their potential in cancer research [92], [96]. DRL was utilized on existing therapeutic methods to build automated radiation adaptation procedures for patients with non-small cell lung cancer (NSCLC). It aims to maximize localized tumor growth control while reducing radiation dose and inhibiting radiation-induced pneumonitis [94]. Furthermore, researchers have explored DRL’s potential for correlating distinct forms of cancer associated with tumor protein 53 (TP53) mutation patterns, and their unique impacts on tumors [100]. Recent developments in advanced quantum DRL have provided a framework for clinical decision-making that can estimate personalized radiotherapy and propose the best dosage adjustment strategy [101]. The implementation of DRL and DL enables the design of sophisticated disease models that takes input from real-world complex data and are not limited to certain pre-described protocols. These trends indicate a rapid adoption of DRL and DL to enhance the potential of AI in cancer research.

## 7 Summary and Outlook

**Fig. 7:**
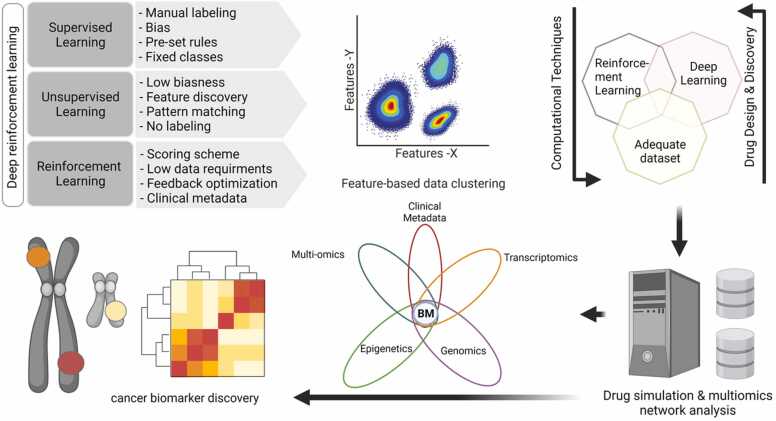
Conventional modeling and deep learning framework for precision medicine and drug discovery. Knowledge from DL and DRL techniques in the presence of adequate datasets can assist in cancer biomarker discovery. The combination of DL and DRL methods shows promising potential to facilitate the development of anti-cancer drugs based on disease profiles to assist precision medication using multi-omics datasets and clinical metadata. BM, biomarker; DL, deep learning; DRL, deep reinforcement learning.

Clinical research is increasingly demonstrating the broad applicability of DL methods, including in cancer diagnosis and therapy. Recent studies have demonstrated the potential of DL-based ensemble models to augment personalized patient treatment strategies based on medical histories and diagnostics. Advancements in high-throughput and large-scale omics assays have generated large volumes of data that can be utilized for biomarker discovery. Furthermore, precision medicine-based therapeutic interventions for cancer have shown promising efficacy when using a DL-based multimodal ensemble strategy that involves gene-protein-metabolite interaction (Fig. 7). These approaches involve *de novo* drug synthesis, candidate gene clustering, and clinical outcome prediction based on a set of biomarkers discovered by processing complex features from multi-omics datasets.

However, DL models have several limitations, including small sample sizes, lack of interpretability, reliability of computational resources, and scarcity of professional expertise [102]. Training a DL model requires large numbers of well-characterized samples, and a small sample size has been shown to decrease the predictability and accuracy of DL models. Furthermore, the quality of the training data is critical because down- or up-sampling of an imbalanced dataset may introduce biases into model predictions. Complex DL models that are applied to large volumes of data, including imaging datasets, must be trained iteratively, which is computationally expensive. In this context, consensus neural networks can provide effective interrogation of data and may aid in reducing features. In other cases, conventional ML methods can be trained faster than DL models [103]. DL-based prediction requires manual curation; thus, the selection and validation of molecular features and signatures using a DL model remain pertinent to biomarker discovery in cancer research. In conclusion, we anticipate that DL and AI have great potential for identifying biomarkers for cancer and in other medical areas as well.

## CRediT authorship contribution statement

**Vivek Bhakta Mathema:** Conceptualization, Writing – original draft. **Partho Sen:** Conceptualization, Writing – original draft. **Santosh Lamichhane:** Writing – original draft. **Matej Orešič:** Writing – original draft. Sakda Khoomrung: Conceptualization, Writing – original draft.

## Declarations of interest

None.

## Glossary

**AI**: artificial intelligence
**ANN**: artificial neural network
**AUROC**: receiver operating characteristic curve
**CDSS**: clinical decision support systems
**CNN**: convolutional neural networks
**CT**: computed tomography
**DESI**: Desorption electrospray ionization
**DL**: deep learning
**DNN**: deep neural networks
**DRL**: Deep reinforcement learning
**GAN**: generative adversarial network
**GCNN**: graph convolutional neural network
**GNN**: graph neural networks
**HCC**: hepatocellular carcinoma
**KRT7**: Keratin 7
**LTMS**: long-short-term memory
**LUAD**: lung adenocarcinoma
**MIBC**: muscle-invasive bladder cancer
**ML**: machine learning
**MRI**: magnetic resonance imaging
**MS**: mass spectrometry
**NSCLC**: non-small cell lung cancer
**RL**: reinforcement learning
**RNN**: recurrent neural networks
**RTART**: recursive partitioning and regression trees
**SL**: swarm learning
**SMILES**: simplified molecular-input line-entry system
**TCGA**: the cancer genome atlas
**TL**: transfer learning
**TP53**: tumor protein 53
